# Physiologic–Inflammatory–Nutrition (TRIAD-TB) Score at 72 Hours Predicts 30-Day Mortality and Length of Stay in Pulmonary Tuberculosis: A Prospective Cohort Study

**DOI:** 10.3390/biomedicines13122901

**Published:** 2025-11-27

**Authors:** Ionut-Valentin Stanciu, Ariadna-Petronela Fildan, Venkata Sai Harshabhargav Chenna, Adrian Cosmin Ilie, Emanuela Tudorache, Ovidiu Rosca, Livia Stanga, Gabriel Veniamin Cozma, Ionela Preotesoiu, Elena Dantes

**Affiliations:** 1 Faculty of Medicine, “Ovidius” University of Constanta, 900470 Constanta, Romania; ionut.stanciu@365.univ-ovidius.ro (I.-V.S.); petronela.fildan@365.univ-ovidius.ro (A.-P.F.); ionela.preotesoiu@365.univ-ovidius.ro (I.P.); elena.dantes@gmail.com (E.D.); 2 Doctoral School of Medicine, “Ovidius” University of Constanta, 900470 Constanta, Romania; 3 Faculty of Medicine, University of Perpetual Help System DALTA, Manila 1740, Philippines; dr.harshabhargav@gmail.com; 4 Department III Functional Sciences, Division of Public Health and Management, “Victor Babes” University of Medicine and Pharmacy Timisoara, 300041 Timisoara, Romania; ilie.adrian@umft.ro; 5 Center for Research and Innovation in Precision Medicine of Respiratory Diseases, “Victor Babes” University of Medicine and Pharmacy Timisoara, Eftimie Murgu Square 2, 300041 Timisoara, Romania; emanuela.tudorache@umft.ro; 6 Methodological and Infectious Diseases Research Center, Department of Infectious Diseases, “Victor Babes” University of Medicine and Pharmacy Timisoara, Eftimie Murgu Square 2, 300041 Timisoara, Romania; ovidiu.rosca@umft.ro; 7 Discipline of Microbiology, Faculty of Medicine, “Victor Babes” University of Medicine and Pharmacy Timisoara, Eftimie Murgu Square 2, 300041 Timisoara, Romania; 8 Thoracic Surgery Research Center, “Victor Babes” University of Medicine and Pharmacy Timisoara, Eftimie Murgu Square 2, 300041 Timisoara, Romania; 9 Department of Surgical Semiology, Faculty of Medicine, “Victor Babes” University of Medicine and Pharmacy Timisoara, Eftimie Murgu Square 2, 300041 Timisoara, Romania

**Keywords:** tuberculosis, pulmonary, hypoxemia, C-reactive protein, serum albumin, body mass index

## Abstract

**Background and Objectives**: Ward-level risk in pulmonary tuberculosis (TB) is often estimated from static admission data. This study evaluated a pragmatic composite—TRIAD-TB—integrating physiology (SpO_2_, respiratory rate), inflammation (systemic immune-inflammation index [SII], C-reactive protein [CRP]), and nutrition (BMI, albumin), augmented by 72 h changes in CRP and albumin, to predict 30-day mortality and hospital length of stay (LOS). **Methods**: A pooled prospective cohort of 126 HIV-negative adults without chronic systemic immunosuppression hospitalized with culture-confirmed pulmonary TB at two Romanian tertiary centers was analyzed. TRIAD-TB combined z-scored admission markers and 72 h deltas. The primary outcome was 30-day all-cause mortality; secondary outcomes included LOS. Associations were estimated using Firth logistic regression (mortality) and quasi-Poisson regression (LOS). Discrimination and overall performance were summarized by AUC and Brier score; internal performance used bootstrap optimism-correction. **Results**: Across TRIAD-TB tertiles, 30-day mortality increased from 2.4% to 16.7%, and mean LOS rose from 24.7 ± 5.8 to 32.1 ± 7.3 days. Each SD increase in TRIAD-TB was associated with higher odds of death (adjusted OR 2.4, 95% CI 1.3–4.8; *p* = 0.006) and longer hospitalization (adjusted IRR 1.19, 95% CI 1.09–1.30; *p* < 0.001). The full model discriminated mortality well (AUC 0.84; Brier 0.067) and explained 21.8% of LOS deviance. Early dynamics were informative: higher CRP ratio (72 h/0 h) and albumin decline tracked with adverse outcomes. An admission-only “mini-TRIAD” retained strong discrimination (AUC 0.79). **Conclusions**: A dynamic composite leveraging routine vitals and laboratory tests—plus 72 h trajectories—accurately stratified short-term risk in hospitalized pulmonary TB, while an admission-only “mini-TRIAD” retained strong discrimination. Together, these tools may support early escalation, targeted monitoring, and capacity planning. TRIAD-TB may support early escalation, targeted monitoring, and capacity planning; however, because it was derived in HIV-negative, non-immunosuppressed adults in an Eastern European setting, TRIAD-TB and the admission-only “mini-TRIAD” require external validation, including in cohorts with substantial HIV co-infection and different comorbidity profiles, before any broader implementation.

## 1. Introduction

Tuberculosis (TB) remains a substantial clinical and organizational burden across Eastern Europe, with Romania persistently reporting among the highest notification rates in the EU/EEA and a marked concentration of disease in vulnerable groups (e.g., people experiencing homelessness, people who use drugs, and those in prisons) [1]. Spatial analyses further suggest heterogeneity in incidence and clustering across Romanian counties, shaped by air pollution and socio-economic gradients, underscoring the need for service models that respond to local epidemiology and surging capacity demands [2]. Within hospital systems, TB admissions are non-trivial contributors to bed occupancy, and Romanian and neighboring health systems have been evaluating shifts from hospital-centric to ambulatory-first care to decompress inpatient units without compromising outcomes [3]. Nevertheless, in-hospital mortality for TB persists—regional cohorts from south-eastern Romania documented measurable inpatient fatality risk, often concentrated early after admission—highlighting a window where timely risk-stratification could alter trajectories [4].

At the bedside, simple physiological signals—oxygen saturation (SpO_2_) and respiratory rate—capture cardiopulmonary strain quickly and inexpensively. Across diverse TB cohorts, early decompensation is frequently heralded by tachypnea and hypoxemia; prediction efforts for inpatient mortality consistently elevate these markers as high-yield features [5,6]. Even outside explicit acute respiratory failure, chronic or persistent hypoxemia among hospitalized adults with TB is common and portends adverse outcomes, reinforcing the salience of pulse oximetry-informed care pathways [7]. These observations motivate physiology-anchored early warning strategies in TB wards.

Inflammation is the second pillar. C-reactive protein (CRP) has been repeatedly linked to bacillary burden and disease activity, and serial CRP declines during early treatment, correlating with clinical response and risk of adverse events, suggesting prognostic value beyond diagnosis alone [8]. While CRP has been evaluated as a triage/screening tool with mixed performance across populations and settings, its biologic responsiveness to treatment makes it attractive for short-horizon monitoring once TB is diagnosed or strongly suspected [9,10,11,12,13,14]. Beyond single analytes, composite cell-count indices derived from the complete blood count—such as the systemic immune-inflammation index (SII)—track myeloid–lymphoid balance and have shown associations with PTB severity, cavity formation, and acute-phase activation, offering analytically cheap surrogates of inflammatory tone [15].

Nutrition is the third, deeply interwoven axis. Malnutrition is highly prevalent at TB presentation and is independently associated with worse treatment outcomes, slower culture conversion, and higher mortality; meta-analytical evidence reinforces an inverse relationship between body mass index (BMI) and TB risk and outcomes [11,12]. In the inpatient setting, serum albumin—reflecting both nutritional reserve and inflammation-driven redistribution—has repeatedly predicted in-hospital death among adults with TB, even after adjustment for age and comorbidities [13,14]. Together, BMI and albumin provide complementary information about energy stores and visceral protein status that is immediate to clinical decision-making. These three domains are tightly interconnected: systemic inflammation can worsen respiratory mechanics and gas exchange through parenchymal injury and immunothrombosis, while the resulting catabolic drive and anorexia aggravate malnutrition, which in turn impairs immune function and respiratory muscle performance. Existing TB risk-stratification tools have largely focused on cross-sectional symptoms and signs or single-timepoint laboratory tests, with limited integration of these interacting axes and minimal incorporation of early dynamic biomarkers that capture treatment response trajectories.

A limitation of most ward-level risk assessments is their static nature—estimating prognosis from admission snapshots while early clinical courses diverge. In TB, meaningful physiologic and inflammatory changes often emerge over the first days of therapy (e.g., improving oxygenation, down-trending CRP, stabilizing vital signs), whereas non-responders remain inflamed, hypoxemic, and catabolic [5,8,9,10]. Capturing these short-horizon trajectories may refine discrimination between improving and non-improving phenotypes and identify patients who warrant escalation, nutrition-first bundles, or closer monitoring.

Against this backdrop, we conceptualized a pragmatic composite—the TRIAD-TB score—that integrates three routinely accessible domains at admission (physiology, inflammation, nutrition) and augments them with 72 h deltas in CRP and albumin. The approach leverages ubiquitous ward data (vital signs, CBC-derived indices, CRP, albumin, BMI) rather than specialized cytokine panels, aiming for immediate implementability in resource-constrained TB units [1,2,3,5,6,7,8,9,11,12,13,14,15]. We selected a 72 h window because prior work shows that CRP can decline substantially over the first 2–3 days of effective TB therapy, and early changes in albumin often accompany shifts in inflammatory tone and fluid balance [8,10]. We hypothesized that (i) higher TRIAD-TB scores would associate with greater 30-day mortality and longer length of stay; (ii) TRIAD-TB would outperform domain-specific comparators built from physiology-only (SpO_2_, RR), inflammation-only (CRP, SII), or nutrition-only (BMI, albumin) inputs; and (iii) incorporating 72 h deltas would add incremental discrimination beyond baseline values alone.

## 2. Materials and Methods

### 2.1. Study Design and Setting

We conducted a prospective observational cohort in two Romanian tertiary centers (Constanța and Timișoara) and analyzed all participants as a single pooled population of patients with TB admitted from November 2022 to September 2025. The protocol, data dictionary, and analysis plan were finalized before data lock. Reporting follows STROBE recommendations for observational cohort studies and, for model specification/performance reporting, TRIPOD elements relevant to derivation studies of prognostic scores [16,17]. Clinical care—including microbiology, laboratory platforms, and nursing escalation criteria—followed national practice aligned with ATS/CDC/IDSA guidance for drug-susceptible tuberculosis [17,18].

### 2.2. Participants, Eligibility, and Case Definitions

Adults (≥18 y) hospitalized with culture-confirmed pulmonary TB were enrolled consecutively. Inclusion required a positive mycobacterial culture from respiratory samples using routine culture methods (solid Löwenstein–Jensen and/or automated liquid systems per site standard) and ≤7 days of anti-TB therapy prior to “time 0” labs (first 24 h of admission). We excluded exclusive extrapulmonary TB, pregnancy, known HIV infection, chronic systemic immunosuppression (≥10 mg prednisolone-equivalent > 1 month), or subjects missing any core TRIAD-TB predictors. Baseline comorbidities were abstracted from charts; diabetes required a documented diagnosis or treatment. These definitions mirror contemporary clinical guidance for drug-susceptible TB programs [18].

Thus, the present derivation cohort consists of HIV-negative adults without chronic systemic immunosuppression, and TRIAD-TB should currently be considered applicable only to this population in health-system contexts broadly similar to our two Romanian tertiary centers. We did not adjust or stratify primary analyses by days on anti-TB therapy within this 0–7-day window because of the limited sample size and event counts; thus, some attenuation or modification of baseline CRP and albumin gradients by prior treatment cannot be excluded.

### 2.3. Measurements and Variable Definitions

Within 24 h, we recorded vital signs (respiratory rate [RR], heart rate, and peripheral oxygen saturation on room air [SpO_2_] by pulse oximetry), demographics, smoking status, and anthropometrics. Body mass index (BMI) was computed as kg/m^2^ and categorized underweight (<18.5), normal (18.5–24.9), and overweight/obese (≥25) using standard clinical cut-points [19]. Venous blood was obtained for the following: complete blood count (CBC) with differentials to compute the systemic immune-inflammation index (SII = platelets × neutrophils/lymphocytes), C-reactive protein (CRP), and serum albumin. Albumin was treated both as a nutrition marker and a negative acute-phase reactant [20]. SII was chosen a priori as a low-cost whole-blood index of innate immune activation, consistent with its original description and later applications as a composite of neutrophils, lymphocytes, and platelets [21]. Given the bedside utility of CRP in TB and the known prognostic value of early CRP kinetics, we protocolized repeat CRP and albumin at 72 ± 6 h after “time 0” [8,10].

CRP, albumin, and CBC-derived indices were measured in the routine clinical laboratories of the two participating tertiary hospitals using site-specific analyzers with standard internal quality control procedures. We did not apply additional cross-laboratory recalibration beyond routine clinical quality assurance; instead, continuous predictors were later z-standardized at the cohort level (Section 2.4) to mitigate center-level scaling differences when constructing TRIAD-TB. Residual inter-laboratory variability in absolute CRP, albumin, or SII values is therefore possible and should be considered when interpreting raw gradients. Repeat CRP and albumin at 72 ± 6 h were protocolized for all enrolled participants; differential counts at 72 h to derive SII were unavailable in eight cases, so analyses involving SII ratio (72 h/0 h) were conducted using complete-case data (*n* = 118).

### 2.4. Score Specification (TRIAD-TB) and Orientation

TRIAD-TB integrates three domains—physiology, inflammation, and nutrition—plus short-horizon (72 h) trajectories. To minimize center-level scaling effects, all continuous inputs were standardized to cohort z-scores (mean = 0, SD = 1). The composite was oriented toward higher risk with worse physiology and inflammation, poorer nutrition, and failure to improve: TRIAD-TB = +z(SII) + +z(CRP) + +z(RR) − z(SpO_2_) − z(BMI) − z(albumin) + z(CRP_72_h/CRP_0_h) + z(albumin_0_h − albumin_72_h). Thus, a lack of CRP fall (ratio ≈ 1) and any early albumin drop increase the score; improvements move it downwards [8,10,21]. TRIAD-TB tertiles were defined using data-driven cut-points at the 33rd and 67th percentiles of the composite distribution, and were used here for descriptive stratification rather than as prespecified clinical thresholds.

This z-score formulation improves internal coherence across centers but is inherently sample-dependent: the absolute TRIAD-TB value will change if the underlying means and standard deviations differ. Consequently, external users would either need to re-standardize the component variables to their own population or re-express the composite as a simplified, integer points-based score derived and calibrated in larger, independent cohorts. In this derivation study, we retained the z-score specification as a pragmatic, transparent starting point.

We deliberately specified TRIAD-TB as an equal-weight sum of its z-standardized components to maximize transparency and reduce the risk of overfitting in a modest derivation sample with few events. In truth, this pragmatic choice does not imply that each domain contributes equally to risk; rather, it provides a simple starting point that can be refined in larger or external cohorts using penalized regression and cross-validation to estimate more flexible weights.

### 2.5. Outcomes

The primary outcome was all-cause 30-day mortality (in-hospital or documented within 30 days of admission). Secondary outcomes were hospital length of stay (LOS, days from admission to discharge), ICU admission during index hospitalization, and time to smear conversion where available. Outcome ascertainment used electronic and paper medical records with cross-checks by a second abstractor on a 10% random sample (discrepancies adjudicated by consensus).

### 2.6. Statistical Analysis

All analyses were two-sided (α = 0.05). Distributional assumptions were screened using Shapiro–Wilk and visual inspection (histograms/QQ-plots); where normality/variance homogeneity was doubtful, we used robust or non-parametric tests consistent with best practice [22,23]. Group comparisons proceeded via Welch ANOVA (or Kruskal–Wallis where appropriate) with Games–Howell post hoc tests when heteroscedasticity was present [22]. Categorical variables used χ^2^ (Yates) or Fisher’s exact as appropriate. Monotonic associations were summarized by Spearman’s ρ. Multicollinearity among predictors was assessed using variance inflation factors, which were <2 for all covariates, indicating low collinearity. Model fit and assumptions were evaluated using deviance and Pearson residuals, leverage diagnostics, and visual inspection of residual plots.

For binary outcomes (30-day mortality) we used Firth penalized logistic regression to mitigate small-sample and rare-event bias and potential separation, reporting adjusted odds ratios (OR) with 95% CIs [24]. For LOS (overdispersed count of days), we modeled expected LOS using quasi-Poisson regression with robust standard errors, an established approach for overdispersed counts; results are presented as incidence rate ratios (IRRs) [25]. Model specification was deliberately parsimonious: the primary exposure was TRIAD-TB (per SD), with age, sex, diabetes, and MDR-TB included as prespecified clinical covariates based on prior evidence, and without additional data-driven variable selection to avoid overfitting in a low-event setting.

For mortality models, we report discrimination by AUC (c-statistic) and overall performance by Brier score; calibration was assessed by calibration slope and visual calibration plots, aligning with contemporary guidance on prediction model evaluation [26,27]. For LOS models, we report explained deviance (quasi-likelihood). Internal performance was estimated via bootstrap optimism-correction (1000 resamples) to reduce overfitting. For both the mortality (Firth logistic) and LOS (quasi-Poisson) models, internal performance estimates (AUC, Brier score, calibration slope, and explained deviance) were optimism-corrected using 1000 bootstrap resamples. Calibration was assessed visually using plots of observed versus predicted probabilities across risk deciles for 30-day mortality. Analyses adhered to key TRIPOD items on reporting model specification, handling of predictors, and performance metrics [17,26,27,28]. Generic early warning scores (EWSs) such as the National Early Warning Score (NEWS) or Modified Early Warning Score (MEWS) were not prospectively calculated, and several required components were incompletely captured in the electronic record; therefore, a formal, patient-level benchmarking of TRIAD-TB against these EWSs was not feasible in the present derivation cohort. All analyses were conducted in R.

## 3. Results

The cohort was predominantly male (100/126, 79.4%) and current smokers were frequent (93/126, 73.8%). The mean age was 51.1 ± 14.9 years and BMI averaged 21.6 ± 3.5 kg/m^2^, with 26.2% underweight and 18.3% overweight/obese. MDR-TB prevalence was 12.7%. Physiologic stress at presentation was notable: mean SpO_2_ on room air was 93.8 ± 2.9%, respiratory rate was 22.1 ± 4.3/min, and heart rate was 96.7 ± 14.2/min. Inflammatory and nutritional markers indicated high disease activity and limited reserves (CRP 74.3 ± 38.6 mg/L; SII 1.5 ± 0.8 × 10^6^/µL; albumin 35.1 ± 5.7 g/L), as seen in Table 1.

Higher TRIAD-TB severity tracked with worse physiology, greater inflammation, and poorer nutrition. From the low → high tertile, SpO_2_ fell from 95.1 ± 2.2% to 92.4 ± 2.6% (Δ −2.7%, *p* < 0.001) and respiratory rate rose from 20.3 ± 3.6 to 24.1 ± 4.2/min (Δ +3.8/min, *p* < 0.001). CRP increased from 55.8 ± 28.4 to 93.2 ± 40.1 mg/L (Δ +37.4 mg/L, *p* < 0.001) and SII from 1.1 ± 0.5 to 1.9 ± 0.9 × 10^6^/µL (Δ +0.8 × 10^6^/µL, *p* < 0.001). Nutritional indices were progressively worse (BMI 22.6 ± 3.4 → 20.6 ± 3.5 kg/m^2^, *p* = 0.012; albumin 37.4 ± 5.0 → 32.8 ± 5.6 g/L, *p* = 0.001), supporting a coherent gradient across domains (Table 2). Because TRIAD-TB tertiles are constructed directly from these same physiologic, inflammatory, and nutritional inputs, the presence of a monotonic gradient across tertiles is mathematically expected and should be interpreted primarily as the internal consistency of the composite rather than independent biological validation.

Early treatment dynamics diverged by baseline severity. The CRP ratio (72 h/0 h) worsened stepwise from 0.62 ± 0.18 in the low tertile to 0.89 ± 0.24 in the high tertile (*p* < 0.001), indicating blunted inflammatory resolution with increasing severity. Albumin rose slightly in low severity (Δ +0.7 ± 1.3 g/L) but declined in high severity (Δ −0.6 ± 1.6 g/L; *p* < 0.001). Similarly, SII improved in low severity (ratio 0.78 ± 0.22) but increased in high severity (1.03 ± 0.27; *p* = 0.002) (Table 3). 72 h CRP and albumin measurements were protocolized for all participants, whereas 72 h CBC with differential to calculate SII was missing in eight cases; Table 3 therefore reports complete-case trajectories for the 118 patients with available 0 h and 72 h SII. Reasons for missing 72 h SII were not systematically recorded and likely reflect a mixture of early discharge, logistic constraints, and early deterioration; accordingly, these dynamic analyses should be interpreted as exploratory.

Adverse outcomes increased monotonically with severity. Thirty-day mortality rose from 1/42 (2.4%) in the low tertile to 7/42 (16.7%) in the high tertile (≈7-fold increase; *p* = 0.018). Mean length of stay lengthened by 7.4 days (24.7 ± 5.8 → 32.1 ± 7.3; *p* < 0.001). ICU admission climbed from 7.1% to 28.6% (*p* = 0.012), and time to smear conversion was prolonged by 8.4 days (26.3 ± 8.1 → 34.7 ± 9.4; *p* < 0.001), as presented in Table 4.

TRIAD-TB correlated moderately with longer hospitalization (ρ = 0.46, *p* < 0.001) and with 30-day mortality on the point-biserial scale (ρ = 0.32, *p* < 0.001). A lower admission of SpO_2_ related to a longer LOS (ρ = −0.39, *p* < 0.001), while a higher respiratory rate correlated with mortality (ρ = 0.28, *p* = 0.002). Early inflammatory and nutritional dynamics were informative: a higher CRP ratio associated with mortality (ρ = 0.29, *p* = 0.001) and increases in albumin over 72 h related to a shorter LOS (ρ = −0.33, *p* < 0.001). SII also tracked with LOS (ρ = 0.31, *p* < 0.001), and lower BMI modestly predicted longer stays (ρ = −0.22, *p* = 0.013), as seen in Table 5.

After multivariable adjustment, each SD increase in TRIAD-TB was associated with more than double the odds of 30-day death (adjusted OR 2.4, 95% CI 1.3–4.8; *p* = 0.006). Age (per 10 years; OR 1.3, 0.9–1.8; *p* = 0.121), male sex (OR 1.1, 0.4–3.1; *p* = 0.84), diabetes (OR 1.6, 0.5–4.7; *p* = 0.388), and MDR-TB (OR 1.9, 0.7–5.3; *p* = 0.204) did not reach conventional statistical significance in this low-event sample; however, their point estimates were directionally consistent with the prior literature, and the wide confidence intervals suggest limited power rather than an absence of effect. Within this context, TRIAD-TB emerged as an independent predictor of 30-day mortality (Table 6) but should not be interpreted as supplanting established risk factors.

**Figure 1:**
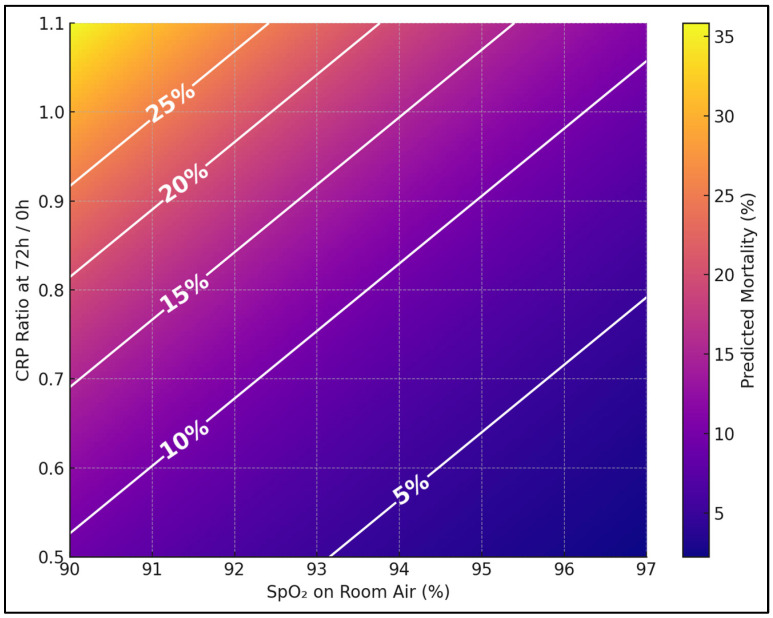
Predicted 3-day mortality by SpO2 and 72 h CRP ratio.

Figure 1 shows predicted 30-day mortality (%) across a clinically realistic grid of admission SpO_2_ (90–97%) and early CRP trajectory (72 h/0 h = 0.5–1.1), with other TRIAD-TB components held at cohort means. Iso-risk contours (5%, 10%, 15%, 20%, 25%) make bedside thresholds obvious—e.g., patients at SpO_2_ 92% with CRP ratio 0.95 sit near the 15–20% band, signaling urgent escalation even if vitals are only modestly abnormal.

TRIAD-TB independently predicted longer hospitalization: per SD-increase, LOS rose by 19% (adjusted IRR 1.19, 95% CI 1.09–1.30; *p* < 0.001). Underweight status conferred a modest excess LOS vs. normal BMI (IRR 1.12, 1.01–1.25; *p* = 0.039), whereas overweight/obesity showed a nonsignificant trend toward shorter stays (IRR 0.93, 0.84–1.02; *p* = 0.091). Age, diabetes, and MDR-TB were not significant predictors in this model (all *p* > 0.10), as seen in Table 7.

The full TRIAD-TB model achieved superior discrimination for 30-day mortality (AUC 0.84, 95% CI 0.75–0.92) with better overall accuracy (Brier 0.067) and near-ideal calibration (slope 0.96), and explained 21.8% of deviance for LOS. This outperformed physiology-only (AUC 0.72; Brier 0.091), inflammation-only (AUC 0.69; Brier 0.098), and nutrition-only (AUC 0.66; Brier 0.104) comparators. A pragmatic “mini-TRIAD” using admission-only inputs retained strong discrimination (AUC 0.79, 95% CI 0.69–0.88; Brier 0.079) and good calibration (0.93), suggesting feasible deployment when 72 h deltas are unavailable (Table 8). The 30-day mortality rate in the cohort was 8.7% (11/126), which provides context for the low Brier scores observed.

**Figure 2:**
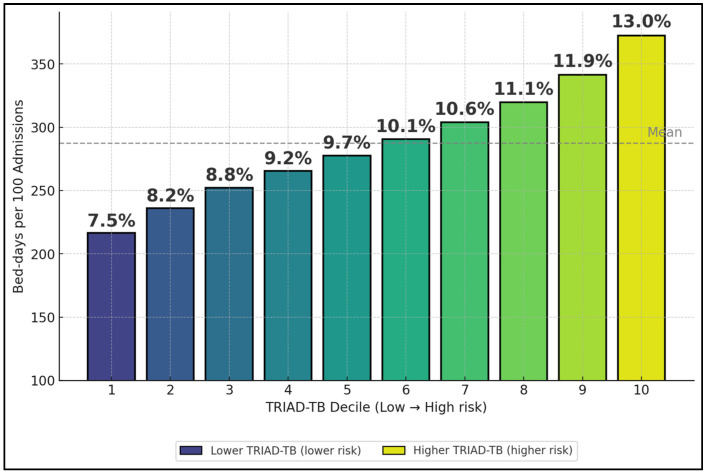
Bed-days concentration across TRIAD-TB deciles. The bars show the proportion of total bed-days (sum of LOS across all admissions) contributed by each decile of TRIAD-TB, illustrating risk concentration in the highest deciles. Values represent point estimates without confidence intervals and are intended for descriptive capacity planning rather than precise inferential comparisons.

Figure 2 translates the LOS IRR into planning terms by estimating bed-days per 100 admissions across TRIAD-TB deciles (low → high risk). The top two deciles consume ~24.9% of bed-days (11.9% + 13.0%), quantifying how targeted protocols for the highest-risk patients can free capacity fastest. Percent share labels (one decimal) are printed above each bar for quick use in ward huddles.

## 4. Discussion

### 4.1. Analysis of Findings

The composite TRIAD-TB construct—integrating physiology, inflammation, and nutrition with short-horizon deltas—aligned with and extended prior tuberculosis (TB) severity instruments that were built chiefly from symptoms and single-timepoint variables. Earlier clinical scores such as the Bandim TBscore and TBscore II predicted adverse outcomes using symptom/functional domains but did not leverage early biomarker dynamics, limiting responsiveness to the first days of treatment when trajectories begin to diverge [29,30]. More recent models, including the TREAT rule and externally validated prognostic scores derived from routine hospital data, similarly focused on admission covariates without capturing near-term change [31,32]. By comparison, adding 72 h CRP and albumin shifts in TRIAD-TB improved discrimination relative to admission-only comparators, a direction consistent with contemporary work showing that parsimonious, objective items such as hypoxemia and lymphocyte counts (AHL score) materially stratified in-hospital mortality risk but still relied on baseline status [33]. In contrast, TRIAD-TB explicitly incorporates early 72 h changes in CRP and albumin, thereby adding a temporal dimension that reflects initial treatment response rather than relying solely on cross-sectional burden at admission. This dynamic specification aligns with the clinical observation that trajectories over the first few days often distinguish improving from non-improving phenotypes more clearly than baseline measurements alone.

Physiologic stress captured by oxygenation and ventilatory drive emerged as a dominant axis, in keeping with the literature from severe TB cohorts. Hypoxemia formed a core element of the AHL score and robustly separated risk groups in prospective development and validation cohorts [33]. Historical ICU series reported high mortality among patients requiring invasive support, underscoring how tachypnea–hypoxemia syndromes track with decompensation; in active pulmonary TB needing mechanical ventilation, case fatality was substantial and increased with gas-exchange derangements and multiorgan strain [34,35,36]. In the present cohort, lower SpO_2_ and a higher respiratory rate correlated with death and longer hospital stay, consistent with these data and reinforcing the use of simple bedside physiology as a high-yield signal embedded within composite prediction.

Inflammation constituted the second pillar. Prior work showed that baseline CRP varied with host and mycobacterial factors and reflected disease activity, while systemic inflammatory profiles (including CRP and cytokines) associated with radiographic severity and bacillary burden [37,38]. Longitudinal studies further demonstrated that incomplete early CRP decline related to persistent culture positivity later in the intensive phase, suggesting that early trajectories carry prognostic information beyond static levels [39]. In elderly pulmonary TB, higher CRP associated with delayed smear conversion, adding a clinically practical bridge between inflammatory tone and time to microbiologic response [40]. The graded increase in 30-day mortality with higher CRP ratio (72 h/0 h) replicated these patterns, supporting short-horizon CRP kinetics as an actionable monitor of treatment response and risk.

The nutritional axis exhibited expected and clinically meaningful effects. Population-based analyses linked underweight status to higher all-cause and TB-specific mortality during treatment, with the strongest effects at the lowest BMI strata and within early death windows [41,42,43]. Albumin, while influenced by inflammation (negative acute-phase behavior), consistently functioned as a pragmatic severity and outcome marker across infectious and critical illness contexts; in HIV-associated TB cohorts, hypoalbuminemia predicted death and tracked with disease activity, supporting its dual informative role in energy reservation and inflammatory redistribution [44]. In this study, lower baseline BMI and albumin—and especially the failure of albumin to rise in the first 72 h—related to prolonged length of stay, mirroring the synergy between catabolic load and inflammatory drive described in prior work.

Early microbiologic dynamics provided an external validity check for the inflammatory and physiologic signals. Multiple cohorts identified factors for delayed smear/culture conversion—older age, higher baseline bacillary load, cavitary disease, diabetes, and extensive radiographic involvement—features that overlapped with higher TRIAD-TB strata [45,46,47]. Reports also connected higher CRP with delayed conversion in older adults, again aligning with this study’s observation that blunted early CRP improvement tracked with worse outcomes and longer hospitalization [40,45]. The concordance across physiologic, inflammatory, nutritional, and microbiologic trajectories suggested that the composite captured a coherent “non-improving” phenotype that has been repeatedly associated with the failure to convert and with downstream morbidity.

The concordance between inflammatory and nutritional trajectories and microbiologic response raises the possibility that simple biologic markers such as serial CRP and albumin could serve as pragmatic surrogate endpoints for microbiologic improvement in future trials or treatment-monitoring protocols. In settings where frequent sputum culture or advanced imaging is not feasible, integrating these trajectories into risk scores such as TRIAD-TB may provide an accessible bridge between host response and pathogen clearance.

Finally, the operational implications of risk concentration were consistent with prior experiences implementing simple TB risk tools for triage and escalation. Studies showed that brief, objective models stratified inpatient risk and could help target enhanced monitoring or bundled interventions to patients most likely to die or to consume disproportionate bed-days [31,32,33]. The concentration of bed-days in the highest TRIAD-TB deciles in this cohort closely paralleled such experiences, indicating that embedding admission-plus-72 h reassessment in ward huddles and care pathways could enable earlier escalation, nutrition-first strategies, and improved discharge planning in settings with constrained capacity. A further operational consideration is how TRIAD-TB relates to generic early warning scores such as NEWS or MEWS, which are widely implemented across acute-care wards. In this derivation study, we could not reconstruct these scores reliably, because several components were either not routinely recorded or were incompletely documented. As such, TRIAD-TB should be viewed as a TB-focused complement to, rather than a replacement for, generic EWS, and formal benchmarking against NEWS/MEWS and other ward-level tools is a key priority for future external validation studies.

Importantly, the reported superiority of the full TRIAD-TB model over single-domain comparators is demonstrated under this equal-weight, pragmatic specification. The relative prognostic contribution of each component may differ in larger or more diverse cohorts, and future work should explore penalized approaches (e.g., LASSO, elastic net) and cross-validated weighting schemes to test the robustness and potential optimization of the composite. Regarding the implications for clinical practice, TRIAD-TB offers a pragmatic, bedside-ready way to identify pulmonary TB inpatients at the highest short-term risk using data every ward already collects (vitals, CBC-derived SII, CRP, BMI, albumin) and their 72 h trajectories. In our cohort, risk rose stepwise across tertiles—30-day mortality from 2.4% to 16.7% and mean LOS from 24.7 ± 5.8 to 32.1 ± 7.3 days—while the full model discriminated well (AUC 0.84; Brier 0.067), and a same-day “mini-TRIAD” retained strong performance when deltas were unavailable (AUC 0.79). Clinically, this supports a two-timepoint strategy: (i) admission triage with the mini-TRIAD to trigger closer monitoring, early ICU outreach, and nutrition consults for high-risk patients; and (ii) a scheduled day-3 “re-stratification” using the full TRIAD-TB, incorporating CRP ratio and albumin change, to detect non-responders and prompt escalation (repeat imaging, search for complications, expedited DST/MDR evaluation, adherence/toxicity checks) and proactive discharge planning.

### 4.2. Study Limitations and Future Implications

This was a two-center Romanian cohort of culture-confirmed pulmonary TB that deliberately excluded adults with HIV co-infection and chronic systemic immunosuppression. As a result, TRIAD-TB is currently supported only in HIV-negative, non-immunosuppressed adults hospitalized with pulmonary TB in similar Eastern European health-system contexts, and its performance in high HIV-burden settings or in patients with other forms of profound immunosuppression remains unknown and requires dedicated validation. Required 72 h labs introduce potential survivorship and availability bias (early deaths/missing draws), and measurement heterogeneity across platforms may affect SII/CRP/albumin comparability. We did not systematically incorporate radiographic severity, smear grade, or comprehensive socio-economic determinants, nor did we benchmark against widely used generic early warning scores. Also, although we compared TRIAD-TB with physiology-only, inflammation-only, and nutrition-only models derived from the same dataset, we were unable to benchmark its performance against widely used generic early warning scores (NEWS, MEWS) because these were not prospectively captured in a reconstructable form. This gap limits immediate operational comparability and underscores the need for external validation cohorts in which generic EWSs are recorded alongside TB-specific composites. By design, TRIAD-TB is defined as an equal-weight sum of z-standardized predictors. While this facilitates parsimony and reduces the risk of overfitting in a modest sample, it also means that the raw scale is not directly portable, and it implicitly assumes that each domain contributes equally to risk. External implementation will require either re-standardization to local distributions or the development of a more intuitive, integer points-based version with weights estimated and calibrated in larger, independent cohorts. In addition, although both participating laboratories followed routine quality assurance procedures, we did not undertake bespoke cross-platform harmonization, so some of the observed raw value gradients may partially reflect center-level assay differences rather than purely biological variation. Moreover, the inclusion of adults who had already received up to seven days of anti-tuberculosis therapy before baseline sampling may have attenuated or distorted admission CRP and albumin levels, as these markers can change rapidly with treatment and fluid balance; we were underpowered to perform robust stratified analyses by treatment duration, and this potential confounding should be considered when interpreting the inflammatory and nutritional gradients. Finally, model thresholds were illustrated but not prospectively tested as actionable triggers.

Beyond these limitations, future work should focus on the prospective, multicenter validation of TRIAD-TB in high-burden settings (including those with substantial HIV co-infection), on implementation studies evaluating its impact when embedded as a real-time decision-support tool, and on digital extensions that allow continuous risk updating as new data accrue during hospitalization. Such efforts, ideally combining TRIAD-TB with generic early warning scores and leveraging penalized or machine-learning approaches in larger datasets, will be necessary to refine thresholds, update calibration, and confirm clinical utility.

## 5. Conclusions

Under an equal-weight, pragmatic specification, a dynamic composite integrating physiology (SpO_2_, RR), inflammation (SII, CRP), and nutrition (BMI, albumin)—augmented by 72 h CRP and albumin trajectories—was independently associated with higher 30-day mortality. These findings support TRIAD-TB as a feasible risk-stratification tool for pulmonary TB wards that could guide early escalation and resource allocation. External validation across diverse epidemiologic contexts, calibration updates, and prospective impact evaluation are warranted before routine adoption, but TRIAD-TB may serve as a foundation for next-generation TB risk models that update dynamically during hospitalization and integrate seamlessly into ward-level decision support.

## Figures and Tables

**Table 1:** Cohort characteristics at admission (*n* = 126).

Variable	Value
Age, years (mean ± SD)	51.1 ± 14.9
Male, *n* (%)	100 (79.4)
BMI, kg/m^2^ (mean ± SD)	21.6 ± 3.5
Underweight/Normal/Overweight–Obese, *n* (%)	33 (26.2)/70 (55.6)/23 (18.3)
Current smoker, *n* (%)	93 (73.8)
Diabetes, *n* (%)	18 (14.3)
MDR-TB, *n* (%)	16 (12.7)
SpO_2_ on room air, % (mean ± SD)	93.8 ± 2.9
Respiratory rate, breaths/min (mean ± SD)	22.1 ± 4.3
Heart rate, beats/min (mean ± SD)	96.7 ± 14.2
CRP, mg/L (mean ± SD)	74.3 ± 38.6
Albumin, g/L (mean ± SD)	35.1 ± 5.7
SII, ×10^6^/µL (mean ± SD)	1.5 ± 0.8

SD, standard deviation; BMI, body mass index; MDR-TB, multidrug-resistant tuberculosis; SpO_2_, peripheral oxygen saturation; CRP, C-reactive protein; SII, systemic immune-inflammation index.

**Table 2:** Admission physiology and labs across TRIAD-TB tertiles (*n* = 42 each).

Marker (Mean ± SD)	Low	Middle	High	*p* (ANOVA/KW)
SpO_2_, %	95.1 ± 2.2	93.8 ± 2.4	92.4 ± 2.6	<0.001
Respiratory rate, /min	20.3 ± 3.6	21.9 ± 4.1	24.1 ± 4.2	<0.001
CRP, mg/L	55.8 ± 28.4	73.9 ± 33.7	93.2 ± 40.1	<0.001
SII, ×10^6^/µL	1.1 ± 0.5	1.5 ± 0.7	1.9 ± 0.9	<0.001
BMI, kg/m^2^	22.6 ± 3.4	21.5 ± 3.4	20.6 ± 3.5	0.012
Albumin, g/L	37.4 ± 5.0	35.1 ± 5.1	32.8 ± 5.6	0.001

TRIAD-TB, composite severity score; SpO_2_, peripheral oxygen saturation; CRP, C-reactive protein; SII, systemic immune-inflammation index; BMI, body mass index; ANOVA/KW, analysis of variance/Kruskal–Wallis.

**Table 3:** Seventy-two-hour trajectories by TRIAD-TB tertile (available *n* = 118).

Variable (Mean ± SD)	Low (*n* = 40)	Middle (*n* = 40)	High (*n* = 38)	*p*
CRP ratio (72 h/0 h)	0.62 ± 0.18	0.76 ± 0.20	0.89 ± 0.24	<0.001
ΔAlbumin, g/L (72 h–0 h)	+0.7 ± 1.3	+0.1 ± 1.4	−0.6 ± 1.6	<0.001
SII ratio (72 h/0 h)	0.78 ± 0.22	0.90 ± 0.26	1.03 ± 0.27	0.002

CRP ratio, C-reactive protein at 72 h relative to baseline; ΔAlbumin, albumin change (72 h−0 h); SII ratio, systemic immune-inflammation index at 72 h relative to baseline; h, hours.

**Table 4:** Clinical outcomes by TRIAD-TB tertile (*n* = 42 each).

Outcome	Low	Middle	High	*p*
30-day mortality, *n* (%)	1 (2.4)	3 (7.1)	7 (16.7)	0.018
LOS, days (mean ± SD)	24.7 ± 5.8	27.9 ± 6.4	32.1 ± 7.3	<0.001
ICU admission, *n* (%)	3 (7.1)	6 (14.3)	12 (28.6)	0.012
Time to smear conversion, days	26.3 ± 8.1	30.2 ± 8.7	34.7 ± 9.4	<0.001

LOS, length of stay; ICU, intensive care unit; TRIAD-TB, composite severity score.

**Table 5:** Spearman correlations with outcomes (*n* = 126; FDR-adjusted *p*).

Pair	ρ	*p*
TRIAD-TB–LOS	0.46	<0.001
TRIAD-TB–30-day mortality (pbis)	0.32	<0.001
SpO_2_–LOS	−0.39	<0.001
RR–Mortality	0.28	0.002
CRP ratio (72 h/0 h)–Mortality	0.29	0.001
ΔAlbumin (72 h–0 h)–LOS	−0.33	<0.001
SII–LOS	0.31	<0.001
BMI–LOS	−0.22	0.013
Albumin–Mortality	−0.24	0.007

ρ, Spearman correlation coefficient; pbis, point-biserial correlation; FDR, false discovery rate; LOS, length of stay; SpO_2_, peripheral oxygen saturation; RR, respiratory rate; CRP, C-reactive protein; SII, systemic immune-inflammation index; BMI, body mass index.

**Table 6:** Logistic regression for 30-day mortality (*n* = 126).

Predictor	Adjusted OR (95% CI)	*p*
TRIAD-TB (per SD)	2.4 (1.3–4.8)	0.006
Age (per 10 years)	1.3 (0.9–1.8)	0.121
Male sex	1.1 (0.4–3.1)	0.84
Diabetes	1.6 (0.5–4.7)	0.388
MDR-TB	1.9 (0.7–5.3)	0.204

OR, odds ratio; CI, confidence interval; SD, standard deviation; MDR-TB, multidrug-resistant tuberculosis; TRIAD-TB, composite severity score.

**Table 7:** Poisson regression for hospital length of stay (*n* = 126).

Predictor	Adjusted IRR (95% CI)	*p*
TRIAD-TB (per SD)	1.19 (1.09–1.30)	<0.001
Underweight vs. Normal BMI	1.12 (1.01–1.25)	0.039
Overweight/Obese vs. Normal	0.93 (0.84–1.02)	0.091
Age (per 10 years)	1.04 (0.99–1.10)	0.162
Diabetes	1.05 (0.96–1.15)	0.286
MDR-TB	1.08 (0.98–1.19)	0.108

IRR, incidence rate ratio; CI, confidence interval; LOS, length of stay; BMI, body mass index; MDR-TB, multidrug-resistant tuberculosis; TRIAD-TB, composite severity score.

**Table 8:** Model performance.

Model	Mortality AUC (95% CI)	Brier	Calibration Slope	LOS Explained Deviance
TRIAD-TB (full)	0.84 (0.75–0.92)	0.067	0.96	21.80%
Physiology-only (SpO_2_, RR)	0.72 (0.61–0.83)	0.091	0.88	12.40%
Inflammation-only (SII, CRP)	0.69 (0.58–0.80)	0.098	0.85	11.60%
Nutrition-only (BMI, albumin)	0.66 (0.55–0.78)	0.104	0.83	9.70%
“Mini-TRIAD” (admission only)	0.79 (0.69–0.88)	0.079	0.93	17.30%

AUC, area under the receiver operating characteristic curve; Brier, Brier score (lower is better); LOS, length of stay; TRIAD-TB, composite severity score; “mini-TRIAD,” admission-only variant of TRIAD-TB.

## Data Availability

The data presented in this study are available on request from the corresponding author.

## References

[1] Munteanu I., Cioran N., van Hest R., Abubakar I., Story A., Chiotan D., de Vries G., Mahler B. (2022). Tuberculosis Surveillance in Romania Among Vulnerable Risk Groups Between 2015 and 2017. Ther. Clin. Risk Manag..

[2] Peptenatu D., Băloi A.M., Andronic O., Bolocan A., Cioran N., Gruia A.K., Grecu A., Panciu T.C., Georgescu L., Munteanu I. (2024). Spatio-Temporal Pattern of Tuberculosis Distribution in Romania and Particulate Matter Pollution Associated With Risk of Infection. Geohealth.

[3] Kelly S.L., Jaoude G.J.A., Palmer T., Skordis J., Haghparast-Bidgoli H., Goscé L., Jarvis S.J., Kedziora D.J., Abeysuriya R., Benedikt C. (2023). Public health benefits of shifting from hospital-focused to ambulatory TB care in Eastern Europe: Optimising TB investments in Belarus, the Republic of Moldova, and Romania. PLoS Glob. Public Health.

[4] Arghir O.-C., Dantes E., Otelea M., Rascu A., Borgazi E., Cambrea S.C. (2018). Eight year survey of tuberculosis in-hospital mortality in the South Eastern part of Romania. Rom. J. Leg. Med..

[5] de Almeida C.P.B., Ziegelmann P.K., Couban R., Wang L., Busse J.W., Silva D.R. (2018). Predictors of In-Hospital Mortality among Patients with Pulmonary Tuberculosis: A Systematic Review and Meta-analysis. Sci. Rep..

[6] Bekele A., Boche B., Anagaw Y.K., Ayenew W., Worku M.C., Geremew D.T., Minwagaw T., Melkamu E., Woldeyohanins A.E., Tefera B.B. (2025). Inventory management performance of essential medicines in public health facilities of Jimma Zone, Southwest Ethiopia. PLoS Glob. Public Health.

[7] Zhao W., Xie X., He T., Zhang J., Liu J. (2024). Study on vertical variation characteristics of soil phosphorus adsorption and desorption in black soil region of Northeast China. PLoS ONE.

[8] Wilson D., Moosa M.S., Cohen T., Cudahy P., Aldous C., Maartens G. (2018). Evaluation of Tuberculosis Treatment Response with Serial C-Reactive Protein Measurements. Open Forum Infect. Dis..

[9] Meca A.D., Turcu-Stiolica A., Bogdan M., Subtirelu M.S., Cocoș R., Ungureanu B.S., Mahler B., Pisoschi C.G. (2022). Screening performance of C-reactive protein for active pulmonary tuberculosis in HIV-positive patients: A systematic review with a meta-analysis. Front. Immunol..

[10] Yoon C., Chaisson L.H., Patel S.M., Allen I.E., Drain P.K., Wilson D., Cattamanchi A. (2017). Diagnostic accuracy of C-reactive protein for active pulmonary tuberculosis: A meta-analysis. Int. J. Tuberc. Lung Dis..

[11] Li A., Yuan S.Y., Li Q.G., Li J.X., Yin X.Y., Liu N.N. (2023). Prevalence and risk factors of malnutrition in patients with pulmonary tuberculosis: A systematic review and meta-analysis. Front. Med..

[12] Franco J.V., Bongaerts B., Metzendorf M.I., Risso A., Guo Y., Peña Silva L., Boeckmann M., Schlesinger S., Damen J.A., Richter B. (2024). Undernutrition as a risk factor for tuberculosis disease. Cochrane Database Syst. Rev..

[13] Matos E.D., Moreira Lemos A.C. (2006). Association between serum albumin levels and in-hospital deaths due to tuberculosis. Int. J. Tuberc. Lung Dis..

[14] Okamura K., Nagata N., Wakamatsu K., Yonemoto K., Ikegame S., Kajiki A., Takayama K., Nakanishi Y. (2013). Hypoalbuminemia and lymphocytopenia are predictive risk factors for in-hospital mortality in patients with tuberculosis. Intern. Med..

[15] Yu Z., Shang Z., Huang Q., Wen F., Patil S. (2024). Integrating systemic immune-inflammation index, fibrinogen, and T-SPOT.TB for precision distinction of active pulmonary tuberculosis in the era of mycobacterial disease research. Front. Microbiol..

[16] von Elm E., Altman D.G., Egger M., Pocock S.J., Gøtzsche P.C., Vandenbroucke J.P., STROBE Initiative (2007). Strengthening the Reporting of Observational Studies in Epidemiology (STROBE) statement: Guidelines for reporting observational, studies. BMJ.

[17] Moons K.G., Altman D.G., Reitsma J.B., Ioannidis J.P., Macaskill P., Steyerberg E.W., Vickers A.J., Ransohoff D.F., Collins G.S. (2015). Transparent Reporting of a multivariable prediction model for Individual Prognosis or Diagnosis (TRIPOD): Explanation and elaboration. Ann. Intern. Med..

[18] Nahid P., Dorman S.E., Alipanah N., Barry P.M., Brozek J.L., Cattamanchi A., Chaisson L.H., Chaisson R.E., Daley C.L., Grzemska M. (2016). Official American Thoracic Society/Centers for Disease Control and Prevention/Infectious Diseases Society of America Clinical Practice Guidelines: Treatment of Drug-Susceptible Tuberculosis. Clin. Infect. Dis..

[19] Nuttall F.Q. (2015). Body Mass Index: Obesity, BMI, and Health: A Critical Review. Nutr. Today.

[20] Don B.R., Kaysen G. (2004). Serum albumin: Relationship to inflammation and nutrition. Semin. Dial..

[21] Hu B., Yang X.R., Xu Y., Sun Y.F., Sun C., Guo W., Zhang X., Wang W.M., Qiu S.J., Zhou J. (2014). Systemic immune-inflammation index predicts prognosis of patients after curative resection for hepatocellular carcinoma. Clin. Cancer Res..

[22] Delacre M., Lakens D., Leys C. (2019). Why Psychologists Should by Default Use Welch’s t-Test Instead of Student’s t-Test. Int. Rev. Soc. Psychol..

[23] Midway S., White J.W. (2025). Testing for normality in regression models: Mistakes abound (but may not matter). R Soc. Open Sci..

[24] Suhas S., Manjunatha N., Kumar C.N., Benegal V., Rao G.N., Varghese M., Gururaj G. (2023). Firth’s penalized logistic regression: A superior approach for analysis of data from India’s National Mental Health Survey, 2016. Indian J. Psychiatry.

[25] Ver Hoef J.M., Boveng P.L. (2007). Quasi-Poisson vs. negative binomial regression: How should we model overdispersed count data?. Ecology.

[26] Steyerberg E.W., Vickers A.J., Cook N.R., Gerds T., Gonen M., Obuchowski N., Pencina M.J., Kattan M.W. (2010). Assessing the performance of prediction models: A framework for traditional and novel measures. Epidemiology.

[27] Van Calster B., McLernon D.J., van Smeden M., Wynants L., Steyerberg E.W. (2019). Topic Group ‘Evaluating diagnostic tests and prediction models’ of the STRATOS initiative. Calibration: The Achilles heel of predictive analytics. BMC Med..

[28] Coxe S., West S.G., Aiken L.S. (2009). The analysis of count data: A gentle introduction to poisson regression and its alternatives. J. Personal. Assess..

[29] Wejse C., Gustafson P., Nielsen J., Gomes V.F., Aaby P., Andersen P.L., Sodemann M. (2008). TBscore: Signs and symptoms from tuberculosis patients in a low-resource setting have predictive value and may be used to assess clinical course. Scand. J. Infect. Dis..

[30] Rudolf F. (2014). The Bandim TBscore--reliability, further development, and evaluation of potential uses. Glob. Health Action.

[31] Bastos H.N., Osório N.S., Castro A.G., Ramos A., Carvalho T., Meira L., Araújo D., Almeida L., Boaventura R., Fragata P. (2016). A Prediction Rule to Stratify Mortality Risk of Patients with Pulmonary Tuberculosis. PLoS ONE.

[32] Nguyen D.T., Graviss E.A. (2018). Development and validation of a prognostic score to predict tuberculosis mortality. J. Infect..

[33] Osawa T., Watanabe M., Morimoto K., Yoshiyama T., Matsuda S., Fujiwara K., Furuuchi K., Shimoda M., Ito M., Kodama T. (2024). Activities of Daily Living, Hypoxemia, and Lymphocytes Score for Predicting Mortality Risk in Patients With Pulmonary TB. Chest.

[34] Lee P.L., Jerng J.S., Chang Y.L., Chen C.F., Hsueh P.R., Yu C.J., Yang P.C., Luh K.T. (2003). Patient mortality of active pulmonary tuberculosis requiring mechanical ventilation. Eur. Respir. J..

[35] Ryu Y.J., Koh W.J., Kang E.H., Suh G.Y., Chung M.P., Kim H., Kwon O.J. (2007). Prognostic factors in pulmonary tuberculosis requiring mechanical ventilation for acute respiratory failure. Respirology.

[36] Kim W.Y., Kim M.H., Jo E.J., Eom J.S., Mok J., Kim K.U., Park H.K., Lee M.K., Lee K. (2018). Predicting Mortality in Patients with Tuberculous Destroyed Lung Receiving Mechanical Ventilation. Tuberc. Respir. Dis..

[37] Brown J., Clark K., Smith C., Hopwood J., Lynard O., Toolan M., Creer D., Barker J., Breen R., Brown T. (2016). Variation in C—Reactive protein response according to host and mycobacterial characteristics in active tuberculosis. BMC Infect. Dis..

[38] Mesquita E.D., Gil-Santana L., Ramalho D., Tonomura E., Silva E.C., Oliveira M.M., Andrade B.B., Kritski A., Rede-TB Study group (2016). Associations between systemic inflammation, mycobacterial loads in sputum and radiological improvement after treatment initiation in pulmonary TB patients from Brazil: A prospective cohort study. BMC Infect. Dis..

[39] Miranda P., Gil-Santana L., Oliveira M.G., Mesquita E.D., Silva E., Rauwerdink A., Cobelens F., Oliveira M.M., Andrade B.B., Kritski A. (2017). Sustained elevated levels of C-reactive protein and ferritin in pulmonary tuberculosis patients remaining culture positive upon treatment initiation. PLoS ONE.

[40] Komiya K., Goto A., Kan T., Honjo K., Uchida S., Takikawa S., Yoshimatsu T., Hiramatsu K., Kadota J.I. (2020). A high C-reactive protein level and poor performance status are associated with delayed sputum conversion in elderly patients with pulmonary tuberculosis in Japan. Clin. Respir. J..

[41] Yen Y.F., Chuang P.H., Yen M.Y., Lin S.Y., Chuang P., Yuan M.J., Ho B.L., Chou P., Deng C.Y. (2016). Association of Body Mass Index With Tuberculosis Mortality: A Population-Based Follow-Up Study. Medicine.

[42] Lai H.H., Lai Y.J., Yen Y.F. (2017). Association of Body Mass Index with Timing of Death during Tuberculosis Treatment. PLoS ONE.

[43] Alvarez-Uria G., Midde M., Pakam R., Naik P.K. (2013). Diagnostic and Prognostic Value of Serum Albumin for Tuberculosis in HIV Infected Patients Eligible for Antiretroviral Therapy: Datafrom an HIV Cohort Study in India. Bioimpacts.

[44] Bisognin F., Amodio F., Lombardi G., Bacchi Reggiani M.L., Vanino E., Attard L., Tadolini M., Re M.C., Dal Monte P. (2019). Predictors of time to sputum smear conversion in patients with pulmonary tuberculosis under treatment. New Microbiol..

[45] Unsematham S., Kateruttanakul P. (2013). Factors predicting sputum smear conversion and treatment outcomes in new smear-positive pulmonary tuberculosis. J. Med. Assoc. Thai..

[46] Güler M., Unsal E., Dursun B., Aydln O., Capan N. (2007). Factors influencing sputum smear and culture conversion time among patients with new case pulmonary tuberculosis. Int. J. Clin. Pract..

[47] Singla R., Osman M.M., Khan N., Al-Sharif N., Al-Sayegh M.O., Shaikh M.A. (2003). Factors predicting persistent sputum smear positivity among pulmonary tuberculosis patients 2 months after treatment. Int. J. Tuberc. Lung Dis..

